# Association between one-session bilateral whole-lung lavage and periprocedural complications in patients with pulmonary alveolar proteinosis: a retrospective cohort study

**DOI:** 10.1186/s13023-023-02691-9

**Published:** 2023-04-20

**Authors:** Xiaohan Xu, Huanrong Qiu, Fei Chen, Yuelun Zhang, Xinlun Tian, Yongjian Liu, Ping Wang, Bo Zhu, Yuguang Huang

**Affiliations:** 1 Department of Anesthesiology, State Key Laboratory of Complex Severe and Rare Diseases, Peking Union Medical College Hospital, Chinese Academy of Medical Sciences, Beijing, China; 2 Department of Anesthesiology, Beijing Friendship Hospital, Capital Medical University, Beijing, China; 3 Department of Anesthesiology, Cancer Hospital, Chinese Academy of Medical Sciences, Beijing, China; 4 Central Research Laboratory, Peking Union Medical College Hospital, Chinese Academy of Medical Sciences, Beijing, China; 5 Department of Respiratory and Critical Care Medicine, State Key Laboratory of Complex Severe and Rare Diseases, Peking Union Medical College Hospital, Chinese Academy of Medical Sciences, Beijing, China; 6 Department of Anesthesiology, Peking Union Medical College Hospital, Chinese Academy of Medical Sciences, Beijing, 100730 PR China

**Keywords:** Whole-lung lavage, One-session, Bilateral, Pulmonary alveolar proteinosis, Complication, Safety

## Abstract

**Background:**

Whole lung lavage (WLL) has been recognized as the most effective therapy of severe pulmonary alveolar proteinosis (PAP). Most centers perform the lavage of each lung in two sessions under general anesthesia at an interval of several days to weeks. Compared with two-session WLL, one-session bilateral sequential WLL only requires general anesthesia once. However, the safety of one-session WLL in PAP patients has not been assessed by large cohort studies. In this study, we aimed to investigate the association between the mode of WLL procedure (one-session or two-session) and the risk of periprocedural complications in PAP patients.

**Methods:**

In this single-center retrospective cohort study, we included adult patients who were diagnosed as PAP and had undergone WLL procedures under general anesthesia from 2000 to 2022. Patients requiring extra-corporeal oxygenation during WLL were excluded. Since some patients received multiple WLL procedures, we considered each procedure in one-session or two-session group as a unique unit in our analysis. The primary outcome was the occurrence of any complications during hospitalization, including termination of WLL procedure due to fluid leakage or refractory hypoxemia, bronchospasm, delayed endotracheal extubation, cardiovascular event, pneumothorax, and fever.

**Results:**

We included a total of 175 WLL procedures (118 patients), with 48 in the two-session group and 127 in the one-session group. Periprocedural complications occurred in 17 (35.4%) and 39 (30.7%) procedures in the two-session and the one-session groups, respectively. The risk of periprocedural complications did not differ significantly between groups, after adjusting the unbalanced confounders in a multivariable model (odds ratio 0.95, 95% confidence interval 0.34 to 2.69, *P* 0.929) or by inverse probability of treatment weighting (odds ratio 0.70, 95% confidence interval 0.30 to 1.54, *P* 0.379). Compared with the two-session WLL group, the one-session WLL group had a shorter postprocedural length of hospitalization and comparable decrease in alveolar-arterial oxygen tension gradient from baseline.

**Conclusions:**

One-session bilateral WLL was not associated with an increased risk of periprocedural complications compared with two-session WLL in PAP patients. Experienced physicians may consider performing one-session WLL in view of the comparable safety and efficacy and potential advantages of saving time.

## Background

Pulmonary alveolar proteinosis (PAP) is a rare disease, with an estimated prevalence of 2.04 to 6.2 cases per million [1]. A deficiency of granulocyte-macrophage colony stimulating factor (GM-CSF) activity leads to an impaired clearance of pulmonary surfactant, which further hampers gas exchange and causes progressive dyspnea in PAP [2]. PAP is divided into primary, secondary, and congenital forms [3]. Primary PAP accounts for more than 90% of adult cases [1].

Although PAP can be treated by non-invasive methods, such as GM-CSF substitution, whole lung lavage (WLL) has been recognized as the most effective therapy of severe PAP [4]. The excessive surfactant material in alveoli is washed out by saline solution during WLL [4]. WLL procedure is performed under general anesthesia. An endotracheal double-lumen tube (DLT) is used to isolate the two lungs, which enables one-lung ventilation (OLV) while the other one is lavaged. A large percentage of PAP patients who require WLL procedures present with respiratory failure, and as a result are at a high risk of developing hypoxemia during OLV.

A global survey indicated that the WLL procedure has not been standardized. 85% of the centers in the survey performed the lavages of each lung in two different sessions separated by days to weeks [5]. Compared with two-session WLL, one-session bilateral sequential WLL only requires one time of general anesthesia, and is therefore believed to be more time-saving and cost-effective [6]. However, the anesthesia management of one-session WLL is more challenging. Since the respiratory function of the first lavaged lung may not fully recover soon, it may be difficult to maintain oxygenation by ventilating this lung while the second lung is being lavaged. Encouragingly, three case-series reports have totally described 48 cases of one-session bilateral WLL, and none of these patients developed major complications [6–8]. However, the safety of one-session WLL has not been assessed by larger cohort studies.

In Peking Union Medical College Hospital (PUMCH), a general tertiary hospital and national consulting center of rare diseases in China, we have performed one-session WLL since 2003, with most of WLL procedures performed in one session in the past 10 years. In this study, we aimed to investigate the association between the mode of WLL procedure (one-session or two-session) and the risk of periprocedural complications in PAP patients. We hypothesized that one-session WLL was associated with an increased risk of periprocedural complications compared with two-session WLL.

## Results

**Fig. 1:**
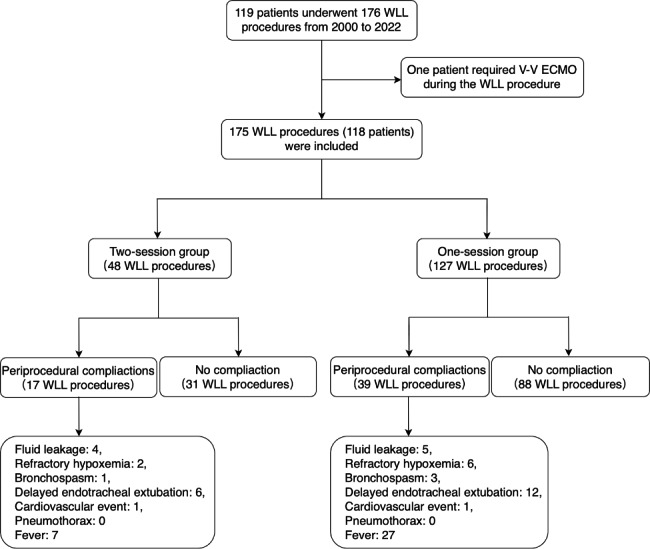
Flowchart of patient selection and description of the primary outcome. Abbreviations: WLL, whole lung lavage; V-V ECMO, veno-venous extra-corporeal membrane oxygenation

There were 119 patients who had undergone 176 WLL procedures under general anesthesia in PUMCH from year 2000 to 2022. One patient was excluded since the patient required venous-venous extra-corporeal oxygenation (ECMO) during the procedure. We included a total of 175 WLL procedures (118 patients), with 48 procedures in the two-session group and 127 procedures in the one-session group (Fig. 1). The mean age of the included patients was 45 ± 11 years, and 67.6% of them were males. 112 patients were diagnosed of primary PAP. During this period of time, 91 patients received WLL once, 15 patients twice, and 12 patients more than twice. The distribution of the following potential confounders differed significantly between the two groups: age, sex, disease severity score (DSS), diffusion dysfunction, preprocedural infection, numbers of previous WLL, surgery year, and anesthesia duration (Table 1).

**Table 1:** Comparison of the potential confounders between the two groups (N = 175)

Variable	Two-session group	One-session group	Standardized mean difference	*P*
Age (year)	47 ± 11	45 ± 11	0.170^a^	0.318
Sex	35 (72.9%)	84 (66.1%)	0.148^a^	0.499
BMI (kg/m^2^)	23.2 ± 3.6	23.4 ± 3.4	0.057	0.734
Smoking	26 (54.2%)	68 (53.5%)	0.013	> 0.999
DSS			0.407^a^	0.124
2	6 (12.5%)	7 (5.5%)		
3	13 (27.1%)	37 (29.1%)		
4	11 (22.9%)	48 (37.8%)		
5	18 (37.5%)	35 (27.6%)		
Diffusion dysfunction			0.493^a^	0.026^a^
1	14 (29.2%)	14 (11.0%)		
2	8 (16.7%)	29 (22.8%)		
3	9 (18.8%)	37 (29.1%)		
4	17 (35.4%)	47 (37.0%)		
Preprocedural infection	1 (2.1%)	31 (24.4%)	0.698^a^	< 0.001^a^
Course of disease (month)	14 [7, 39]	24 [8, 48]	0.080	0.430
Number of previous WLLs	0 [0, 1]	0 [0, 1]	0.113^a^	0.213
Surgery year^b^	4 [2, 6]	11 [9, 16]	1.814^a^	< 0.001^a^
Anesthesia duration (min)	397 ± 97	326 ± 79	0.805^a^	< 0.001^a^
Saline input (L)^c^	20.0 [16.0, 24.5]	20.0 [17.4, 24.0]	0.042	0.996

Abbreviations: BMI, body mass index; DSS, disease severity score; WLL, whole lung lavage. Continuous data were expressed as mean ± standard difference (normally distributed data) or median [interquartile range] (non-normally distributed data), and categorical data were described as number (percentage). Age, BMI, and anesthesia duration were analyzed by Student’s t test. Course of disease, number of previous WLLs, surgery year, and saline input were analyzed by Man-Whitney U test. Sex, smoking, DSS, and diffusion dysfunction were analyzed by Chi-square test. Preprocedural infection was analyzed by Fisher’s exact test. ^a^Standardized mean difference ≥ 0.1 or *P* < 0.05. ^b^Years since 2000. ^c^Total volume of saline input of both lungs during a WLL procedure

Periprocedural complications occurred in 17 (35.4%) and 39 (30.7%) procedures in the two-session and the one-session groups, respectively. Among all the included patients, WLL procedures were terminated 9 times due to fluid leakage, and 8 times due to refractory hypoxemia. Bronchospasm, delayed endotracheal extubation, cardiovascular events, and fever occurred 4, 18, 2 and 34 times, respectively. There was no case of pneumothorax in neither group (Fig. 1). The univariable generalized estimating equation (GEE) model did not show a significant association between periprocedural complication and the group [odds ratio (OR) 0.81, 95% confidence interval (CI) 0.40 to 1.63, *P* 0.552]. Also, the risk of periprocedural complications did not differ significantly between groups, after adjusting the unbalanced confounders in the GEE Model 1 (OR 0.95, 95% CI 0.34 to 2.69, *P* 0.929), or controlling for the most clinically important confounders in GEE Model 2 (OR 0.71, 95% CI 0.34 to 1.49, *P* 0.366) (Table 2). When using inverse probability of treatment weighting (IPTW), we also did not detect a significant association between complications and the group (OR 0.70, 95% CI 0.30 to 1.54, *P* 0.379).

**Table 2:** Generalized Estimating Equation Models of the Primary Outcome (N = 175)

Variable	Model 1			Model 2		
	OR	95% CI	*P*	OR	95% CI	*P*
Group	0.95	0.34 to 2.69	0.929	0.71	0.34 to 1.49	0.366
DSS	1.07	0.67 to 1.71	0.784	1.22	0.87 to 1.72	0.252
Preprocedural infection	2.07	0.86 to 5.01	0.107	1.67	0.72 to 3.87	0.232
Age (10 years)	0.90	0.64 to 1.26	0.523	NA		
Sex	0.44	0.21 to 0.90	0.024^a^	NA		
Diffusion dysfunction	1.18	0.79 to 1.75	0.428	NA		
Number of previous WLLs	0.96	0.79 to 1.16	0.643	NA		
Surgery year^b^	0.98	0.91 to 1.07	0.702	NA		
Anesthesia duration (30 min)	1.16	1.03 to 1.31	0.024^a^	NA		

Abbreviations: OR, odds ratio; CI, confidence interval; BMI, body mass index; DSS, disease severity score; WLL, whole lung lavage; NA, not applicable. ^a^*P*<0.05. ^b^Years since 2000. Model 1 included factors that were unbalanced between the one- and two-session groups. Model 2 included the most clinically important confounders

The postprocedural length of stay (LOS) were 12 [6, 16] days and 5 [3, 8] days in the two-session group and the one-session group, respectively. The one-session group had significantly shorter postprocedural LOS than the two-session group (OR 0.13, 95% CI 0.03 to 0.52, *P* 0.004) after adjusting the confounding effects of unbalanced confounders (Table 3).

**Table 3:** Generalized Estimating Equation Models of the Secondary Outcomes

Variable	Postprocedural LOS (N = 175)			Postprocedural Ventilation Support (N = 175)			Intraprocedural SpO_2_ Nadir (N = 175)			Decrease of P_A−a_O_2_ (N = 166)^a^		
	OR	95% CI	*P*	OR	95% CI	*P*	OR	95% CI	*P*	OR	95% CI	*P*
Group	0.13	0.03 to 0.52	0.004^b^	0.32	0.10 to 1.04	0.058	0.35	0.11 to 1.13	0.080	0.21	0.02 to 1.84	0.157
Age (10 years)	0.95	0.72 to 1.26	0.723	1.14	0.75 to 1.71	0.545	1.15	0.81 to 1.63	0.440	0.59	0.34 to 1.05	0.072
Sex	0.44	0.20 to 0.98	0.044	1.18	0.46 to 3.06	0.734	0.43	0.19 to 0.98	0.044^b^	0.77	0.19 to 3.16	0.717
DSS	1.73	0.92 to 3.23	0.087	1.26	0.65 to 2.46	0.500	0.76	0.38 to 1.51	0.428	1.38	0.45 to 4.23	0.575
Diffusion dysfunction	0.85	0.53 to 1.39	0.527	0.95	0.54 to 1.67	0.861	1.00	0.67 to 1.49	0.996	1.13	0.47 to 2.71	0.776
Preprocedural infection	4.40	1.10 to 17.65	0.036	1.87	0.73 to 4.80	0.196	6.22	2.82 to 13.71	< 0.001^b^	0.36	0.06 to 2.31	0.281
Number of previous WLLs	1.20	0.88 to 1.62	0.248	1.08	0.89 to 1.31	0.416	0.82	0.66 to 1.03	0.084	0.77	0.37 to 1.62	0.492
Surgery year^b^	0.89	0.80 to 0.99	0.030^b^	1.31	1.18 to 1.44	< 0.001^b^	1.07	0.98 to 1.17	0.133	1.08	0.93 to 1.26	0.291
Anesthesia duration (30 min)	0.99	1.07 to 1.35	0.920	1.06	0.89 to 1.23	0.558	1.08	0.93 to 1.26	0.301	0.79	0.63 to 0.99	0.037^b^

Abbreviations: LOS, length of stay; P_A−a_O_2_, alveolar-arterial oxygen tension gradient; SpO_2_, pulse oxygen saturation; OR, odds ratio; CI, confidence interval; BMI, body mass index; DSS, disease severity score; WLL, whole lung lavage. ^a^ Data of P_A−_aO_2_ was missing in nine included whole lung lavage procedures. ^b^*P*<0.05. ^c^Years since 2000

Ventilation support was required after 9 (18.8%) procedures in the two-session group and 45 (35.4%) procedures in the one-session group. Mask ventilation, bilevel positive airway pressure (BIPAP) ventilation, and invasive mechanical ventilation were required after 1, 2, and 6 two-session WLLs, and 18, 15, and 12 one-session WLLs, respectively. However, the occurrence of requiring postprocedural ventilation support did not differ significantly between the two groups (OR 0.32, 95% CI 0.10 to 1.04, *P* 0.058) after controlling the confounding effects (Table 3).

The intraprocedural pulse oxygen saturation (SpO_2_) nadir were 90% [85%, 92%] and 90% [87%, 93%] in the two-session group and the one-session group, respectively. There was no significant difference of intraprocedural SpO_2_ nadir between groups (OR 0.35, 95% CI 0.11 to 1.13, *P* 0.080) in the multivariable model (Table 3).

Data of alveolar-arterial oxygen tension gradient (P_A−a_O_2_) were missing in three two-session WLL procedures and six one-session WLL procedures. P_A−a_O_2_ were decreased by 12.4 [6.3, 23.0] mmHg and 11.5 [2.3, 18.5] mmHg in the two-session group and the one-session group after WLL procedures, respectively. We did not detect a significant difference between the two groups in terms of the decrease of P_A−a_O_2_ (OR 0.21, 95% CI 0.02 to 1.84, *P* 0.157) (Table 3).

## Discussion

This single-center retrospective study indicated that one-session WLL was not associated with a higher risk of periprocedural complications compared with two-session WLL. We also found that the one-session WLL group did not show an increased risk of requiring postprocedural ventilation support or lower intraprocedural SpO_2_ nadir, but had a shorter postprocedural LOS.

As reported by a global survey, fever, hypoxemia, wheezing, pneumonia, and fluid leakage are the five most common complications of WLLs [5]. Fever was also the most frequent occurrence in our cohort. Fever was possibly resulted from infections or stress responses. PAP patients were susceptible to pulmonary infections [2]. We would administrate antibiotics in presence of evidence of postprocedural infections, and none of our febrile patients developed uncontrolled infection. The one-session group tended to have longer procedure than the two-session group (Table 1), and thus may have stronger stress responses. This may explain why the incidence of fever was higher in the one-session group.

Delayed endotracheal extubation was the second common complication in our study. Notably, we defined delayed extubation as leaving operating rooms without extubation. Actually, most of these patients were successfully extubated in respiratory intensive care unit (RICU) within 12 h, which was consistent with the previous reports [5, 6, 9]. Sometimes we did not extubate a patient right after WLL procedures as we waited a spontaneous recovery of neuromuscular blockade given that neostigmine was not effective in reversing deep blockade [10]. This was more likely to occur in the one-session group because of longer anesthesia duration and larger doses of muscle relaxants required (Table 1). However, this problem has been solved since the new reversal agent, sugammadex, became available for clinical use in 2019. Additionally, the one-session group did not show a higher risk of requiring ventilation support following WLL, either (Table 3). All of our patients were discharged without supplemental oxygen or only needing nasal cannulas.

The incidence of fluid leakage ranked the third among WLL complications in our cohort. Fluid leakage is mainly caused by the malposition of DLT, and can lead to respiratory insufficiency of the ventilated lung. There was a high risk of DLT migration when we placed the patient from supine to lateral position and vice versa; thus, it was necessary to check DLT position via a bronchoscopy after each change of position. Other teams recommended to use a supine position to avoid intraprocedural position change [9].

Refractory hypoxemia is another common reason for the termination of WLL procedures. Actually, we did not terminate WLL even when SpO_2_ dropped to 80% temporarily in patients without neurological or cardiovascular comorbidity, given that PAP patients could well tolerate long-term hypoxemia in daily life. Our experience indicated that patients had a high risk of having hypoxemia when placed in lateral decubitus position with the non-dependent lung ventilated due to the ventilation-perfusion mismatch [11]. When saline flowed in, saline compressed the pulmonary capillary bed of the nonventilated lung and shifted the blood flow to the ventilated lung, which ameliorated hypoxemia. Conversely, shunt fraction was increased when saline was drained out, which exacerbated hypoxemia [12]. For patients in the one-session group, refractory hypoxemia often happened during the lavage of the second lung, possibly because the gas exchange function of the already lavaged lung was impaired due to the absorption of saline. Therefore, it was important to suction and re-expand the first lavaged lung, optimize positive end-expiratory pressure (PEEP), and apply diuretics. Using these methods, the one-session group did not have a lower intraprocedural SpO_2_ nadir than the two-session group (Table 3).

Bronchospasm and cardiovascular events did not frequently occur in our cohort. Bronchospasm might result from the stimulation of bronchus by saline. All of the bronchospasm cases in our study were successfully treated by increasing anesthesia depth and administrating methylprednisolone and/or aminophylline. In terms of cardiovascular events, one patient in the two-session group who had pre-existing coronary artery disease developed postprocedural myocardial ischemia. A patient with PAP secondary to *Mycobacterium avium* infection in the one-session group presented severe hypotension and bradycardia during WLL, which possibly resulted from hypoxemia and were reversed by vasopressors. There was no case of pneumothorax in our study. We routinely maintained an airway peak inspiratory pressure below 30 cmH_2_O to prevent pneumothorax during WLL.

The LOS was significantly longer for the two-session group in our study primarily because the patients stayed in hospital for at least one week to wait for the second session after the first session was completed. This further indicated that one-session WLL had greater advantages in terms of saving time and possibly cost as well. The change in P_A−a_O_2_ from baseline did not differ significantly between the two groups, indicating a comparable efficacy of one-session WLL with two-session WLL.

Our study has the following strengths. First, to the best of our knowledge, this was the largest cohort of PAP patients who had undergone WLL. The sample size allowed us to quantitively assess the safety of one-session WLL by comparing the risk of perioperative complications between the two-session and the one-session groups. Second, we attempted to control the confounding effects in a retrospective study framework. In light of the fact that physicians might select patients with better respiratory function to perform one-session WLL, we included DSS score, diffusion dysfunction, and disease course as potential confounders in the multivariable model. Furthermore, as noted before, most of the two-session WLLs included in the study were performed in earlier years, while the one-session WLL procedures have mostly been performed in more recent years. Thus, we adjusted the confounding effect of surgery years. After taking all of the aforementioned factors into consideration, our negative results were not attributed to the differences of patients’ respiratory function at baseline or the improvements of physicians’ skills over time. Third, we included both major complications, such as cardiovascular events, and minor complications, such as bronchospasm and fever. In this way, we could assess the safety of one-session WLL comprehensively.

Our study was limited in the following aspects. First, we should not draw causal conclusions from this observational study. Second, a limited statistical power may lead to false negative results, especially for the marginally insignificant results, such as requiring postprocedural ventilation support and intraprocedural SpO_2_ nadir. Given the rarity of PAP, expanding the sample size from a single center will take a long time; hence a collaboration of multicenter study is warranted. Third, our single-center study only included adult patients; therefore, our conclusions should not be broadly generalized for pediatric patients with congenital PAP or to other centers where physicians might not have rich experience in managing OLV. Fourth, considering the limited number of the patients who developed complications, including all the unbalanced confounders into the multivariable models might compromise the reliability of coefficients. That was why we built Model 2 that only included the most clinically important confounders. Finally, we could not investigate the long-term complication or efficacy of one-session WLL based on existing data.

## Conclusions

In conclusion, we found that one-session bilateral WLL was not associated with an increased risk of periprocedural complications compared with two-session WLL in PAP patients. Our findings suggested that experienced physicians may consider performing one-session WLL in view of the comparable safety and efficacy and potential advantages of saving time.

## Methods

We performed a single-center retrospective cohort study at PUMCH. Our research protocol was approved by the institutional review board of PUMCH (reference number: S-K2095) on May 17, 2022. The requirement of written informed consent was waived by the ethics committee due to the retrospective nature of this study. This manuscript adheres to the STROBE guidelines.

### Study population

We consecutively included adult patients who were diagnosed as PAP and had undergone WLL procedures under general anesthesia from January 1, 2000 to January 31, 2022 in PUMCH. We excluded the patients who had required ECMO during WLL procedures.

Patients who presented with hypoxemia and “crazy paving pattern” on thoracic computed tomography (CT) were suspected of PAP, and would receive bronchoalveolar lavage (BAL). The diagnosis of PAP was based on the presence of periodic acid-Schiff-positive material in and around alveolar macrophages in BAL fluid and/or transbronchial lung biopsies [13]. For patients diagnosed with PAP, the indications for WLL included significant limitation of daily activity due to dyspnea, partial pressure of arterial oxygen < 65 mmHg on room air, P_A−a_O_2_ > 40 mmHg or shunt fraction > 10% [14]. Given that some patients received multiple WLL procedures, we therefore, considered each procedure as a unique unit in our analysis. The two-session group received WLL procedures in which each lung was lavaged in separate sessions, and the one-session group received WLLs in which bilateral lungs were lavaged in one session.

### Variable definition

The primary outcome was the occurrence of one or more periprocedural complications during hospitalization, including termination of WLL due to fluid leakage or refractory hypoxemia, bronchospasm, delayed endotracheal extubation, cardiovascular events, pneumothorax, and fever. In case of fluid leakage or refractory hypoxemia, we would check the position of DLT, suction remaining fluid, adjust ventilation parameters, apply recruitment maneuvers, and perform intermittent two-lung ventilation (TLV) [9]. If these attempts failed, we would consider terminating the procedure. Bronchospasm was identified in presence of significantly increased airway pressure and wheezing on chest auscultation. After completion of WLL procedures, if we could not wean a patient from mechanical ventilation within an hour, we would change the DLT to a single lumen tube and transfer the patient to RICU. This was defined as delayed endotracheal extubation. Cardiovascular events included myocardial ischemia, heart failure, arrhythmia, and cardiac arrest.

The secondary outcomes included postprocedural LOS, postprocedural ventilation support, intraprocedural SpO_2_ nadir, and the decrease of P_A−a_O_2_. For the two-session group, patients stayed in hospital during the interval between the two sessions, and postprocedural LOS was the time from the first session to discharge. Postprocedural ventilation support included non-rebreather mask ventilation, Venturi mask ventilation, BIPAP ventilation, and invasive mechanical ventilation. Decrease of P_A−a_O_2_ was the difference between the preprocedural baseline P_A−a_O_2_ and postprocedural P_A−a_O_2_ at discharge.

We selected the following 12 potential confounders: age, sex, body mass index (BMI), smoking, DSS score [1], diffusion dysfunction, preprocedural infection, course of disease, numbers of previous WLL, surgery year, anesthesia duration, and saline input. Smoking was defined as having a history of cigarette smoking. Patients were asked to quit smoking at least four weeks before scheduled WLLs. The severity of diffusion dysfunction was graded ordinally from 1 to 4 by the 25%, 50%, and 75% quantiles of the P_A−a_O_2_ on room air as thresholds. For patients in whom P_A−a_O_2_ data were unavailable, we used the diffusing capacity of the lungs for carbon monoxide (DL_CO_) instead, which was graded from high to low by quantiles. Preprocedural infection was defined as having active respiratory tract infection within two months before WLL, and infection was usually well controlled before WLL. Course of disease was measured as the time from the onset of PAP-related symptoms to the procedure date. Anesthesia duration was the duration between the induction of general anesthesia and the discharge from the operating room. For patients in the two-session group, anesthesia duration was the sum of the aforementioned duration of each WLL session. Saline input was the total volume of saline infusion to both lungs during a WLL procedure.

### Anesthesia management and procedure description

Intraprocedural monitoring included electrocardiography, invasive arterial blood pressure, SpO_2_, capnograpy, tidal volume, airway pressure, arterial blood gas, and urine output. General anesthesia was induced with a target-controlled infusion of propofol (at effect-site concentration of 4 to 6 mcg/ml) and bolus injection of fentanyl 1.5 to 2 mcg/kg and rocuronium 0.6 to 0.9 mg/kg; and maintained by continuous infusion of propofol and remifentanil, and intermittent bolus injection of fentanyl and rocuronium. We placed a left-sided DLT after anesthesia induction and confirmed the position through a flexible bronchoscopy. The appropriate size of DLT was selected based on the diameters of the trachea and the left mainstem bronchus measured on the thoracic CT. Air leak was checked by immersing the lumen tube orifice of the non-ventilated lung into water while ventilating the other lung. Lung protective ventilation strategy was used in a volume-controlled mode with a PEEP of 5 to 10 cmH_2_O. The tidal volume (4 to 8 ml/kg) and the respiratory rate (10 to 16 bmp) were adjusted to make the end-tidal CO_2_ within a range of 35 to 45 mmHg. The inspired oxygen fraction (FiO_2_) was set to 100% for 15–20 min for de-nitrogenation.

We first lavaged the most severely affected lung, as evidenced by thoracic CT. If both lungs were equally affected, the left lung was lavaged first. The DLT lumen of the lavaged lung was attached to a Y connector. The lavage branch (input circuit) of the Y connector was connected to a warm (37^0^ C) saline bag hung 50 cm above the mid-axillary line, and the drainage branch (output circuit) was connected to a drainage container. The patient was placed in a lateral decubitus and reverse Trendelenburg position with the lavaged lung down. The DLT lumen of the dependent lung was clamped to allow for degassing and hypoxic pulmonary vasoconstriction, while the non-dependent lung was ventilated by 100% oxygen. Fifteen minutes later, the input circuit of the Y connector was open, while the output circuit clamped. The dependent lung was infused with saline in 0.5 to 1 L aliquots, and a chest wall percussion was performed either manually or using a device for 5 min. Afterwards, the patient was placed in a Trendelenburg position with the input circuit clamped and the output circuit open, and the saline was drained by gravity. The input and output volume of saline was recorded. We would suspect fluid leakage if there were large differences between input and output or bubbles popping up in the DLT lumen of the ventilated lung. This cycle was repeated until the returned saline became clear. After the final cycle, we placed the patient in a supine position and suctioned the lavaged lung thoroughly. Then we resumed TLV, and performed recruitment maneuvers. The same algorithm was applied to the contralateral lung. At the end of WLL procedures, we administrated furosemide 5 to 20 mg intravenously to prevent pulmonary edema. Finally, the patient was extubated in the operating room or transferred to RICU intubated.

### Statistical analysis

Continuous data were expressed as mean ± standard difference (normally distributed data) or median [interquartile range] (non-normally distributed data), and categorical data were described as number (percentage). We used the standardized mean difference and statistical tests to compare the differences of the potential confounders between the two groups. Student’s t test and Man-Whitney U test were used to compared the normally distributed and non-normally distributed variables, respectively. Chi-square test or Fisher’s exact test was used to compared the categorical variables where appropriate. A standardized mean difference ≥ 0.1 or a *P* > 0.05 indicated a significant difference. Considering that some patients received multiple WLLs, GEE models were used to analyze the association between the group and the outcomes with a robust standard error estimate in which a random-effects intercept was set among different WLL procedures in the same individual. GEE logistic models were used to study binary outcomes (periprocedural complication and ventilation support). Continuous outcomes were categorized and analyzed using GEE models for clustered ordinal responses. Postprocedural LOS and decrease of P_A−a_O_2_ were categorized by quantiles, and intraprocedural SpO_2_ nadir by 95%, 90%, 85%, and 80% as thresholds. We used two methods to adjust the confounding effects in the analysis of the primary outcome. First, we built two multivariable models. Model 1 included the confounders that were unbalanced between the two groups, and Model 2 included the most clinically important confounders. Second, the IPTW method was used, in which unbalanced cofounders were included to calculate the propensity score. We only built Model 1 in the analysis of secondary outcomes.

For statistical power analysis, probability of the type I error was set to two-sided 0.05. The primary outcome occurred in 17/48 and 39/127 procedures in the two-session group and the one-session group, respectively, providing 80% statistical power to detect an OR larger than 4.15 or smaller than 0.39.

A two-sided *P* value of less than 0.05 indicated statistical significance. Statistical analysis was conducted using R (version 4.1.2, R Foundation for Statistical Computing, Vienna, Austria, 2022) along with the tableone [15], EpiR [16], and geepack [17] packages.

## Data Availability

The datasets used and/or analyzed during the current study are available from the corresponding author on reasonable request.

## Abbreviations

WLL: whole lung lavage
PAP: pulmonary alveolar proteinosis
OR: odds ratio
CI: confidence interval
DLT: double-lumen tube
OLV: one-lung ventilation
PUMCH: Peking Union Medical College Hospital
GEE: generalized estimating equation
IPTW: inverse probability of treatment weighting
ECMO: extra-corporeal membrane oxygenation
CT: computed tomography
BAL: bronchoalveolar lavage
P_A−a_O_2_: alveolar-arterial oxygen tension gradient
TLV: two-lung ventilation
PEEP: positive end-expiratory pressure
FiO_2_: inspired oxygen fraction
RICU: respiratory intensive care unit
LOS: length of stay
SpO_2_: pulse oxygen saturation
BIPAP: bilevel positive airway pressure
BMI: body mass index
DSS: disease severity score
ICC: intraclass correlation coefficient
